# Editorial Note—Special Issue on Pandemic Influenza Vaccination

**DOI:** 10.3390/vaccines14090783

**Published:** 2026-09-08

**Authors:** Francisco Nogareda, Shoshanna Goldin

**Affiliations:** 1 Special Programme Comprehensive Immunization, Pan American Health Organization (PAHO)/World Health Organization (WHO), Washington, DC 20037, USA; 2 World Health Emergencies Programme, World Health Organization (WHO), 1211 Geneva, Switzerland

Influenza pandemics occur when a novel influenza virus strain emerges that spreads rapidly between people with little or no immunity. The risk of an influenza pandemic is real and ongoing. In particular, the potential for zoonotic spillover, urban crowding, and global travel make influenza pandemics unpredictable but not unexpected. 

Medical countermeasures, including vaccines and antivirals, are essential tools for mitigating the health and economic impact of pandemic emergencies. During the 2009 and COVID-19 pandemics, mass national vaccination campaigns were implemented to protect vulnerable populations from severe illness and death, reduce the impact of the pandemic, and achieve higher rates of immunity across the population.

Both timely access to vaccines and experience with reaching adult populations can enable countries to rapidly deploy vaccines during a future pandemic. Preparing for the next influenza or other respiratory pathogen pandemics is a collaborative undertaking that requires whole-of-government and whole-of-society approaches involving a wide range of stakeholders. In this Special Issue, we examine the continuum of pandemic influenza vaccines and vaccination through twelve publications: 

1. The article by Lilley et al. documents that seasonal influenza vaccination provided cross-protection against some, but not all, swine influenza A viruses. Depending on the lineage and clade of swine influenza A viruses, seasonal influenza vaccines may potentially provide some measure of protection while the strain-specific strain undergoes development and production.

2. McCarron et al.’s quantitative analysis of COVID-19 pandemic vaccination coverage and deployment timelines showcases the value of seasonal influenza vaccination programs for pandemic preparedness and response. COVID-19 vaccine roll-out for health workers was faster and more complete, with coverage 2.5 times higher, in countries with a mature seasonal influenza vaccination program. By maintaining and strengthening seasonal influenza vaccination programs, countries could protect health workers and the general public against epidemics and pandemics. 

3. Barratt et al.’s article analyzes operational readiness of long-term care facilities for pandemic vaccination by examining the use of seasonal influenza vaccination. Residents of long-term care facilities are particularly vulnerable during influenza epidemics and pandemics. Seasonal influenza vaccination offers protection to residents and caretakers and serves as a mechanism to establish and strengthen preparedness. 

4. Chadwick et al.’s article provides insight into sustainability factors for influenza vaccine production in low- and middle-income countries. Recognizing the need for more diversified influenza vaccine production for pandemic preparedness and response, the Global Action Plan for Influenza Vaccines (2006–2016) supported manufacturers in establishing influenza vaccine production capacity. Ten years later, this article summarizes the role of cohesive policies, evidence-based public health priorities, robust investment, regulatory readiness, and demand generation in maintaining local production of influenza vaccines. 

5. Taaffe et al.’s article examines the barriers to and enablers of next-generation influenza vaccine R&D. Using a mixed-methods approach, Taaffe documents the challenges posed by scientific, regulatory, and financial constraints. In addition, the article highlights the role that next-generation influenza vaccine development played in supporting rapid COVID-19 vaccine development, underscoring the importance of this work in pandemic preparedness. 

6. Kennedy et al.’s review of the A(H5) vaccine strain update process offers learnings from a vaccine manufacturer’s journey to rapidly respond to the H5N1 zoonotic outbreak. The review explains the steps in duplicating an existing licensed zoonotic vaccine with a modified antigen, using one of the WHO-recommended candidate vaccine viruses that involved the coverage of circulating viruses and possessed rapid availability. This review provides valuable experiences that can guide future zoonotic and pandemic influenza vaccine strain updates. 

7. Bright’s perspective on next-generation influenza vaccine platforms showcases the opportunities and challenges that recent technology advancements offer for a rapid, global pandemic response. The overview of platform speed, clinical performance, manufacturing adaptability, regulatory pathways, and global access provides a strong case for the value of sustained investment in mRNA and other next-generation influenza vaccine products. 

8. Zhang et al.’s perspective on research priorities for zoonotic and pandemic influenza vaccines summarizes the evidence and recommendations of the WHO Public Health Research Agenda for Influenza (2024 update). Informed by systematic literature reviews, expert consultations, and an analysis of learnings from recent health emergencies, the research agenda provides a roadmap for zoonotic and pandemic influenza vaccine preparedness.

9. Morgan and Mazur’s perspective considers opportunities to strengthen global pandemic preparedness. The review features three actions: establishing predictable demand for seasonal influenza vaccination, incentivizing research and development in pandemic influenza vaccines, and diversifying influenza vaccine platforms to amplify global production capacity. The perspective highlights opportunities for manufacturers, countries, and global stakeholders to advance pandemic influenza vaccine security during the interpandemic period.

10. Delfino et al.’s article examines lessons from past pandemics and global strategies to identify the challenges and opportunities for sustainable manufacturing capacities and equitable access. This article focuses on Argentina’s participation in the mRNA Technology Transfer Program, co-led by the World Health Organization and Medicines Patent Pool, as a model for strengthening local production capacity and contributing to pandemic preparedness.

11. Chadwick et al. developed a second article that used a mixed-methods approach (e.g., surveys and interviews of influenza vaccine technology transfer recipients) to identify learnings and opportunities for next-generation influenza vaccines. This article also documented the spillover benefits from influenza vaccine technology transfer experiences that supported COVID-19 and routine vaccine development. 

12. Rawlings et al.’s perspective piece discusses the Pandemic Influenza Preparedness Framework as a model for efficient and equitable pandemic response. This article highlights that the World Health Organization, through the Pandemic Influenza Preparedness Framework, has secured 11% of estimated global pandemic influenza vaccine production, enabling timely access for developing countries.

This collection of articles covers a wide spectrum of topics on pandemic influenza vaccination, examining the role of research and development, manufacturing and market-shaping, opportunities to sustain local production of seasonal and pandemic products, and preparedness for a future pandemic influenza vaccine response. In addition, this issue provides insight into national capacities for deployment of pandemic influenza vaccines and the value of seasonal influenza vaccination in providing a foundation for pandemic response.

## Data Availability

No new data were created or analyzed in this study. Data sharing is not applicable to this article.

